# Supplementary figures and images for: Pharmacological inhibition of lysine-specific demethylase 1 (LSD1) induces global transcriptional deregulation and ultrastructural alterations that impair viability in Schistosoma mansoni
Source: PLoS Negl Trop Dis. 2020 Jul 1;14(7):e0008332. doi: 10.1371/journal.pntd.0008332 (PMC7329083; doi:10.1371/journal.pntd.0008332)

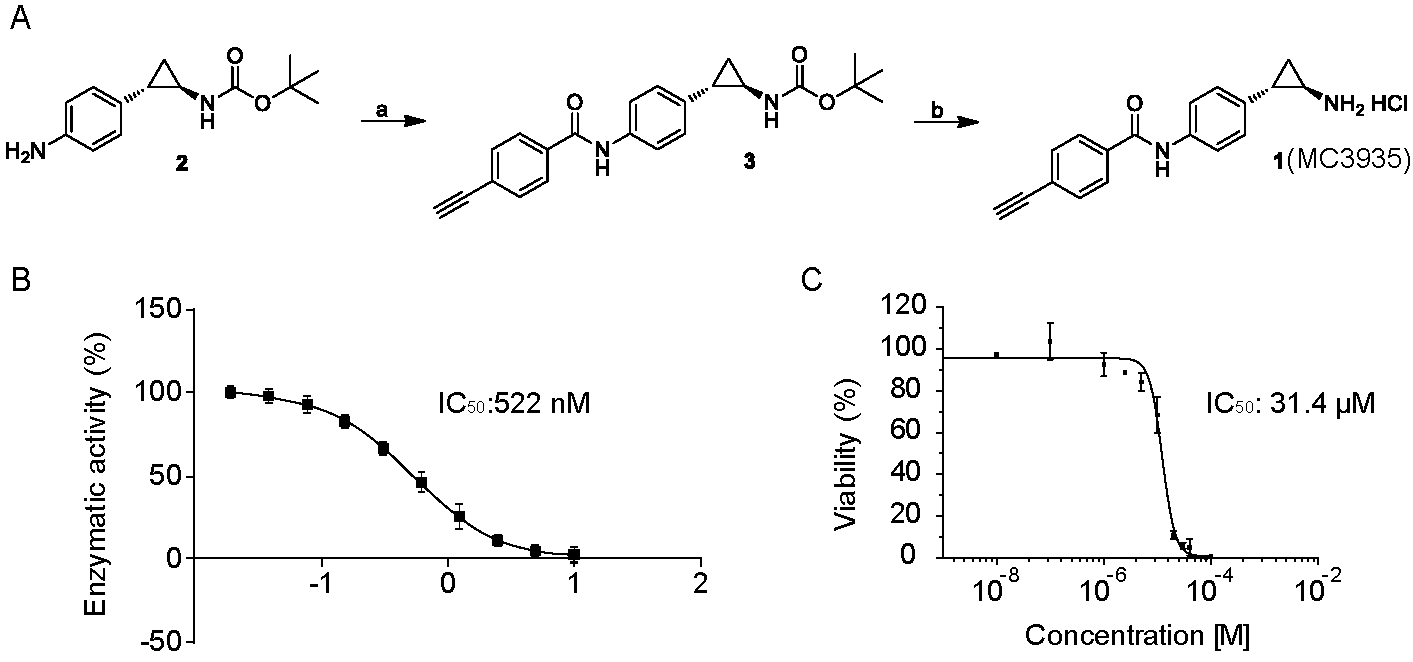

Supplement: S1 Fig — (A). Compound 1 (MC3935) was synthesized by coupling racemic tert-butyl (trans-2-(4-(4-ethynylbenzamido)phenyl)cyclopropyl) carbamate 2, prepared as previously reported,1 with the commercially available 4-ethynylbenzoic acid followed by acidic deprotection of the Boc protected amine 3. Reagents for the synthesis of compound 1 (MC3935): (a) HOBt, EDCI, TEA, dry DMF, rt; (b) HCl 4N in dioxane, dry THF, 0°C-rt. (B). MC3935 inhibits the catalytic activity of recombinant human LSD1 (hLSD1). The concentration required to inhibit the activity of the purified hLSD1 protein by 50% (IC50) is shown in the graph. (C). Schistosomula viability is impaired by MC3935 treatment. The concentration required to cause mortality in 50% of the parasites (IC50) after 48 hours is shown in the graph. (TIF) [file pntd.0008332.s001.tif]

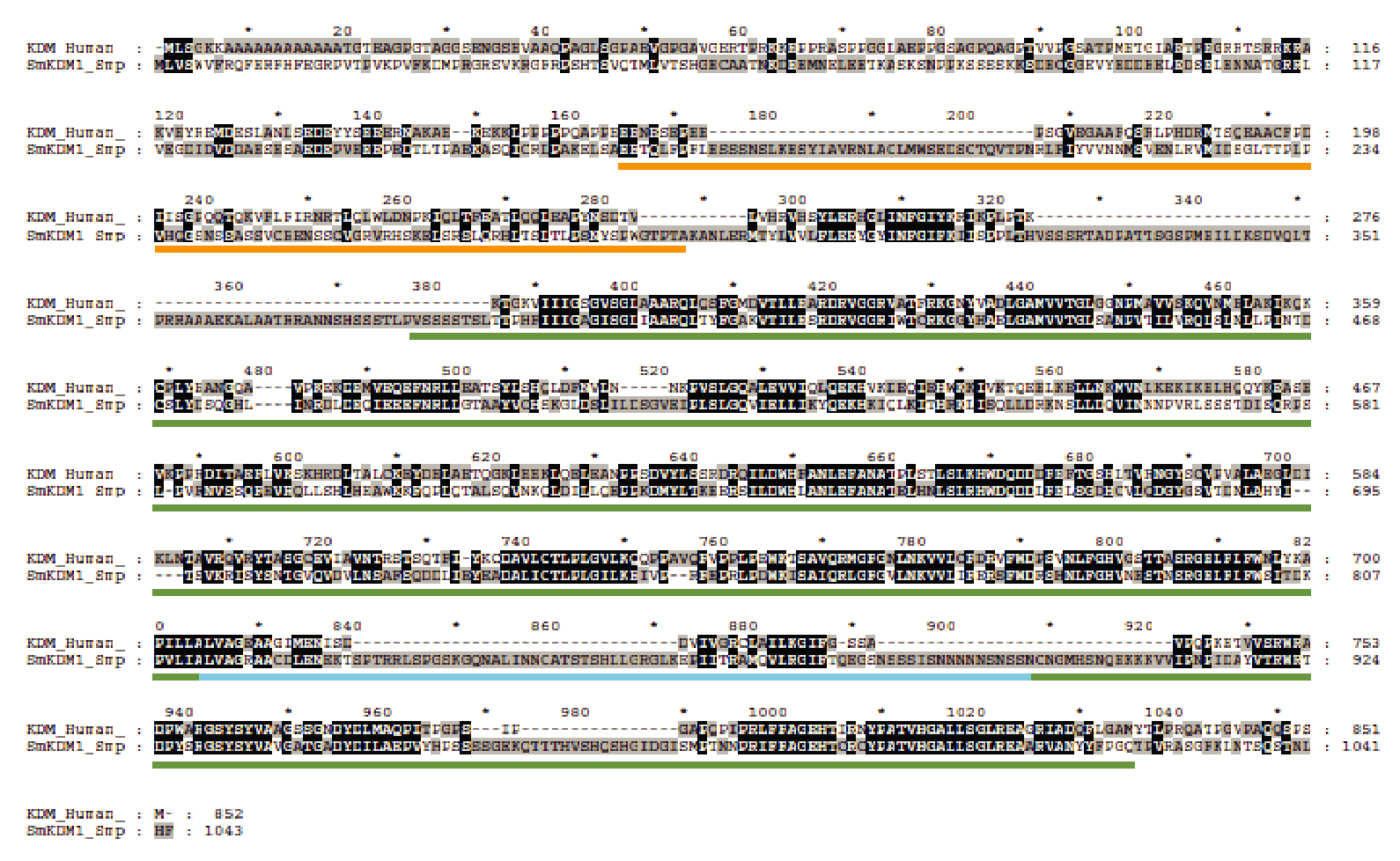

Supplement: S2 Fig — Sequence alignment using the Clustal Omega tool was performed including the Homo sapiens—NP_055828, and Schistosoma mansoni–XP_018652619.1. The functional domains of the LSD1 protein family are underlined as follows: the SWIRM domain in orange (165–287 aa), the amino-oxidase-like domain in green (379–824 and 909–1136 aa) and the TOWER domain in blue (825–908 aa). Amino acid positions refer to the SmLSD1 protein. Unique amino acid sequences found within the SmLSD1 polypeptide are shown as dashes. (TIF) [file pntd.0008332.s002.tif]

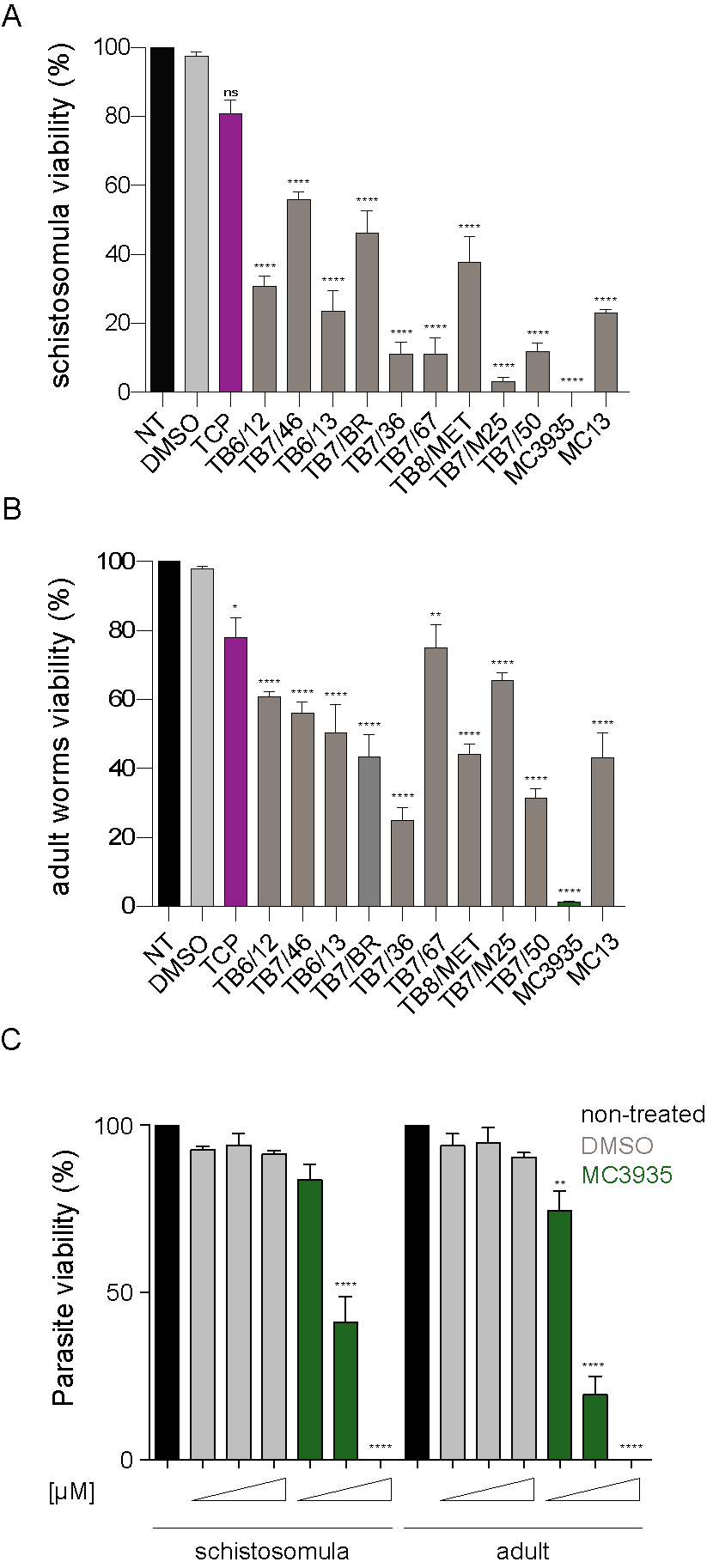

Supplement: S3 Fig — Twenty thousand schistosomula (A) or ten adult worm pairs (B) were incubated with 25 μM of LSD1 inhibitors or DMSO (nontreated parasites were included as an additional control) and submitted to an ATP cell viability assay. Tranylcypromine (TCP, red bars) is a well-known irreversible LSD1 inhibitor. Twelve different compounds based on the TCP scaffold were tested. (C) Dose-dependent toxicity of MC3935 (at 1, 10 or 25 μM) on schistosomula or adult worm pairs. Incubation times for schistosomula and adult worms were 72 h and 96 h, respectively. The results of three independent assays are shown; error bars represent the SD. Statistical significance, comparing treated and vehicle conditions, was determined using one-way ANOVA, with *p<0.05, **p<0.01 and ****p<0.0001, and ns standing for non-significant. (TIF) [file pntd.0008332.s003.tif]

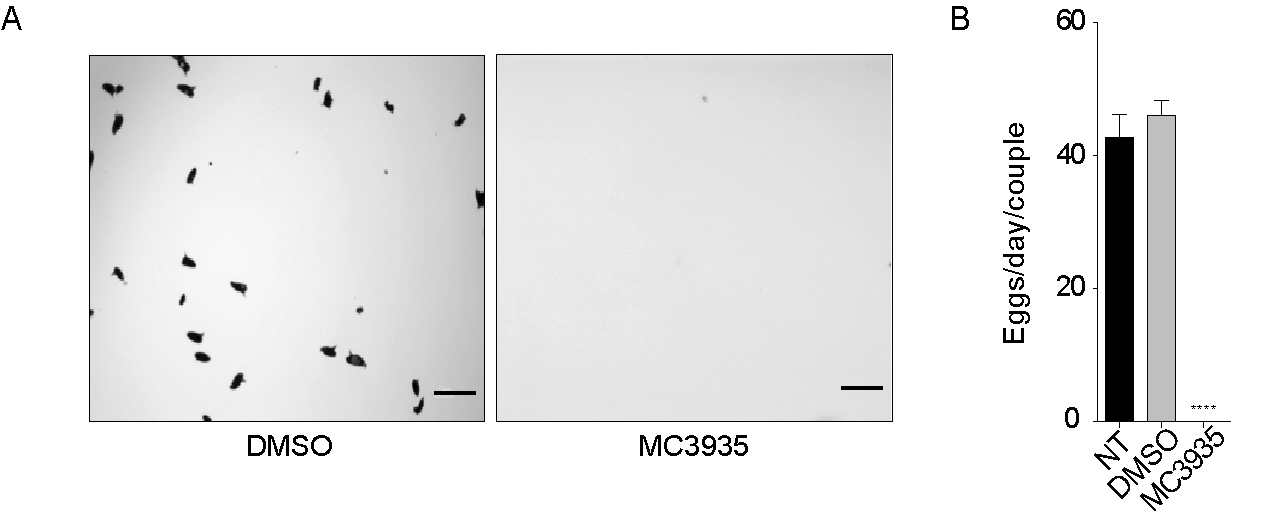

Supplement: S4 Fig — (A). Adult worm pairs were treated (or not, NT) with 0.25% DMSO or 25 μM MC3935 and cultivated for 96 h. The number of laid eggs was counted daily and a representative image was recorded. Scale bar: 250 μm. (B). Quantification of eggs normalized by the number of adult worm pairs and days of treatment. Statistical significance, comparing MC3935-treated and vehicle conditions, was determined using Student´s t-test, with ****p<0.0001. (TIF) [file pntd.0008332.s004.tif]

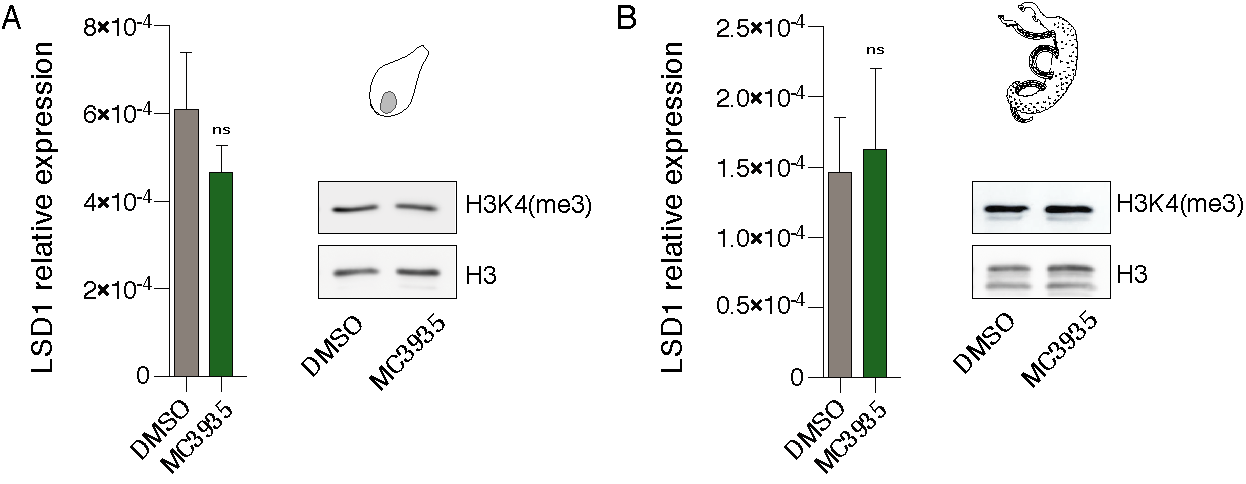

Supplement: S5 Fig — Quantitative RT-PCR analysis of SmLSD1 mRNA or western blot analyses of SmLSD1 protein from schistosomula (A) or adult worms (B) after 72 h or 96 h incubation time with MC3935, respectively. The bars indicate standard deviations from three independent measurements. Histone H3 was included in western blots as the loading control. Statistical significance, comparing MC3935-treated and vehicle conditions, was determined using Student´s t-test, with ns standing for non-significant. (TIF) [file pntd.0008332.s005.tif]

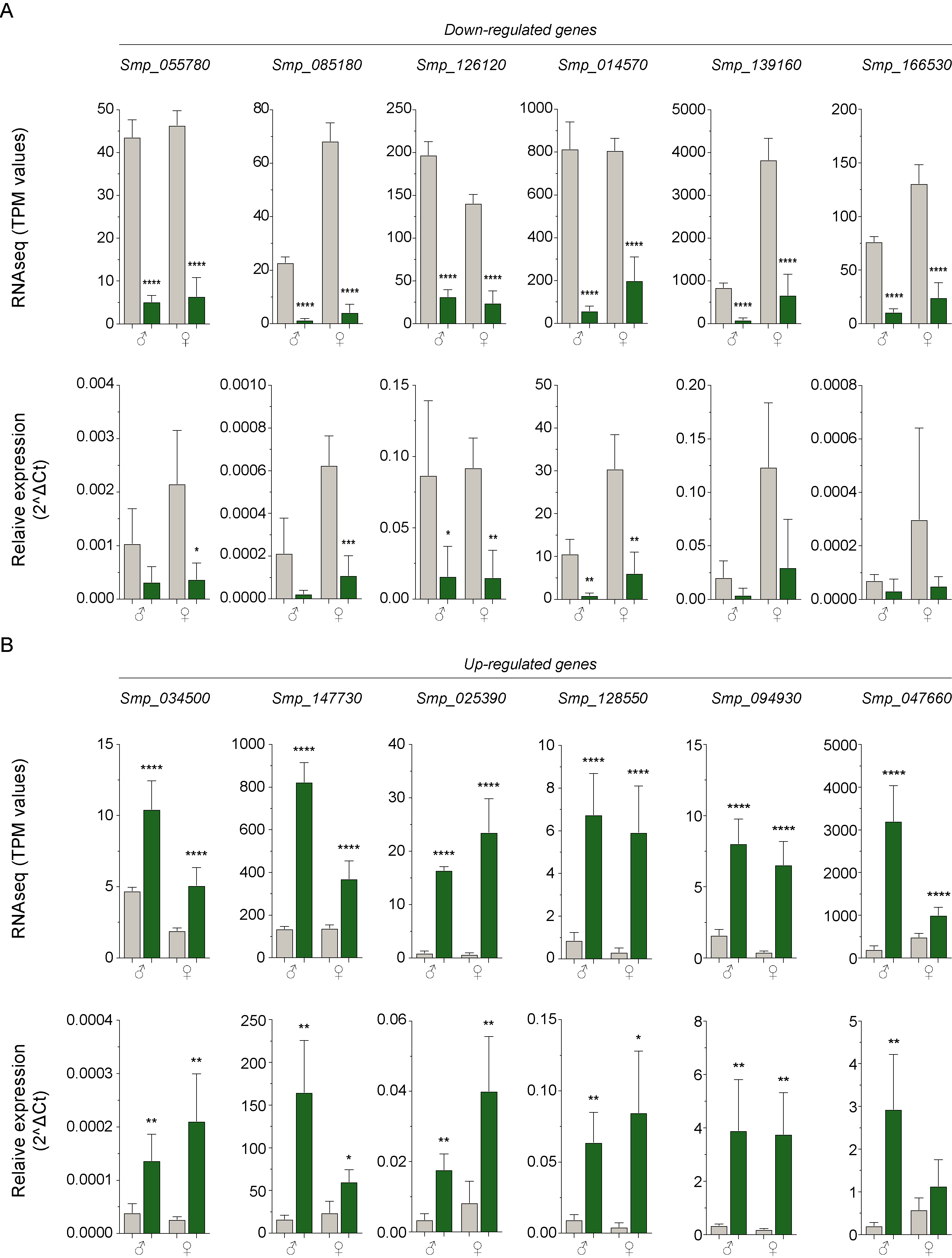

Supplement: S6 Fig — The expression of twelve selected genes was measured by qRT-PCR in RNA samples extracted from male or female parasites exposed for 48 h in vitro either to DMSO (gray bars) or 25 μM of MC3935 (green bars). Top panels in A or B show the validation of down-regulated or up-regulated genes, respectively, from samples submitted to RNA-Sequencing (TPM values). Bottom panels in A or B show the validation of down-regulated or up-regulated genes, respectively, from samples submitted to cDNA synthesis (Relative expression 2⌃ΔCt). Expression was normalized as indicated in the Methods, and the lowest normalized value among the control biological replicates was chosen as reference and arbitrarily set to 1. Relative expression of all other control and treated samples was calculated in relation to that value. Graphs show the mean +_ S.D. of four biological replicates for each condition in males and females. Statistical significance, comparing MC3935-treated and vehicle conditions, was evaluated with the Student´s t-test and significant changes are marked by asterisk with *p<0.05, **p<0.01, ***p<0.001 and ****p<0.0001. Down-regulated genes: Smp_055780: smdr2; Smp_085180: cathepsin B (C01 family); Smp_126120: LAMA protein 2; Smp_014570: Saposin1; Smp_139160: SmCL2 peptidase (C01 family); Smp_166530: phospholipase A. Up-regulated genes: Smp_034500: Dual specificity protein phosphatase 10; Smp_147730: single Kunitz protease inhibitor; Smp_025390: putative calcium dependent protein kinase; Smp_128550: src type protein tyrosine kinase; Smp_094930: early growth response protein 1; Smp_047660: ferritin2C heavy polypeptide 1. (TIF) [file pntd.0008332.s006.tif]

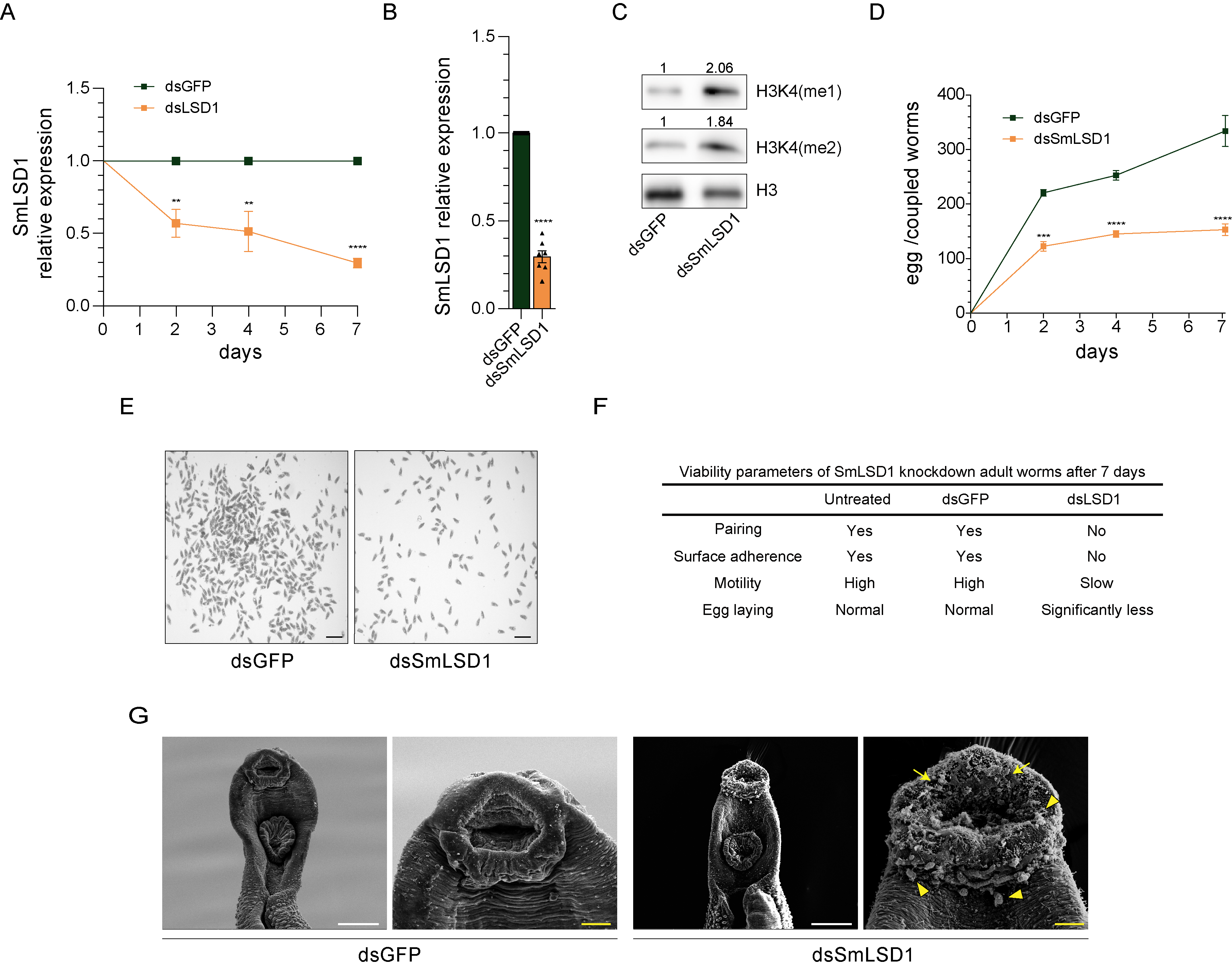

Supplement: S7 Fig — (A). Adult worm pairs were soaked with 30 μg of dsDNA and cultivated for up to 7 days. A silencing of 70% was obtained for SmLSD1 mRNA at day 7 (panels A and B). (C) Western blot analysis of total protein extracts from GFP- or SmLSD1-silenced worms at day 7. Band intensity quantifications (obtained with Image J) are shown above each image, and they were normalized by the H3 band. (D and E) Egg production by GFP- or SmLSD1-silenced female worms was monitored daily (scale bar = 200 μm). (F) Several parameters for adult worm viability were monitored daily using a light microscope, until day 7. The viability parameters were reviewed and scored by two independent observers. (G) SmLSD1 RNAi-mediated phenotypic effects observed by scanning electron microscopy. Arrows point to fissures and arrowheads to blisters in the oral sucker of male worms (scale bar = white (5 μm) and yellow (1 μm). Statistical significance was determined using one-way ANOVA, with **p<0.01, ***p<0.001 and ****p<0.0001. The images displayed are representative of three independent experiments, with approximately 20 samples analyzed. (TIF) [file pntd.0008332.s007.tif]

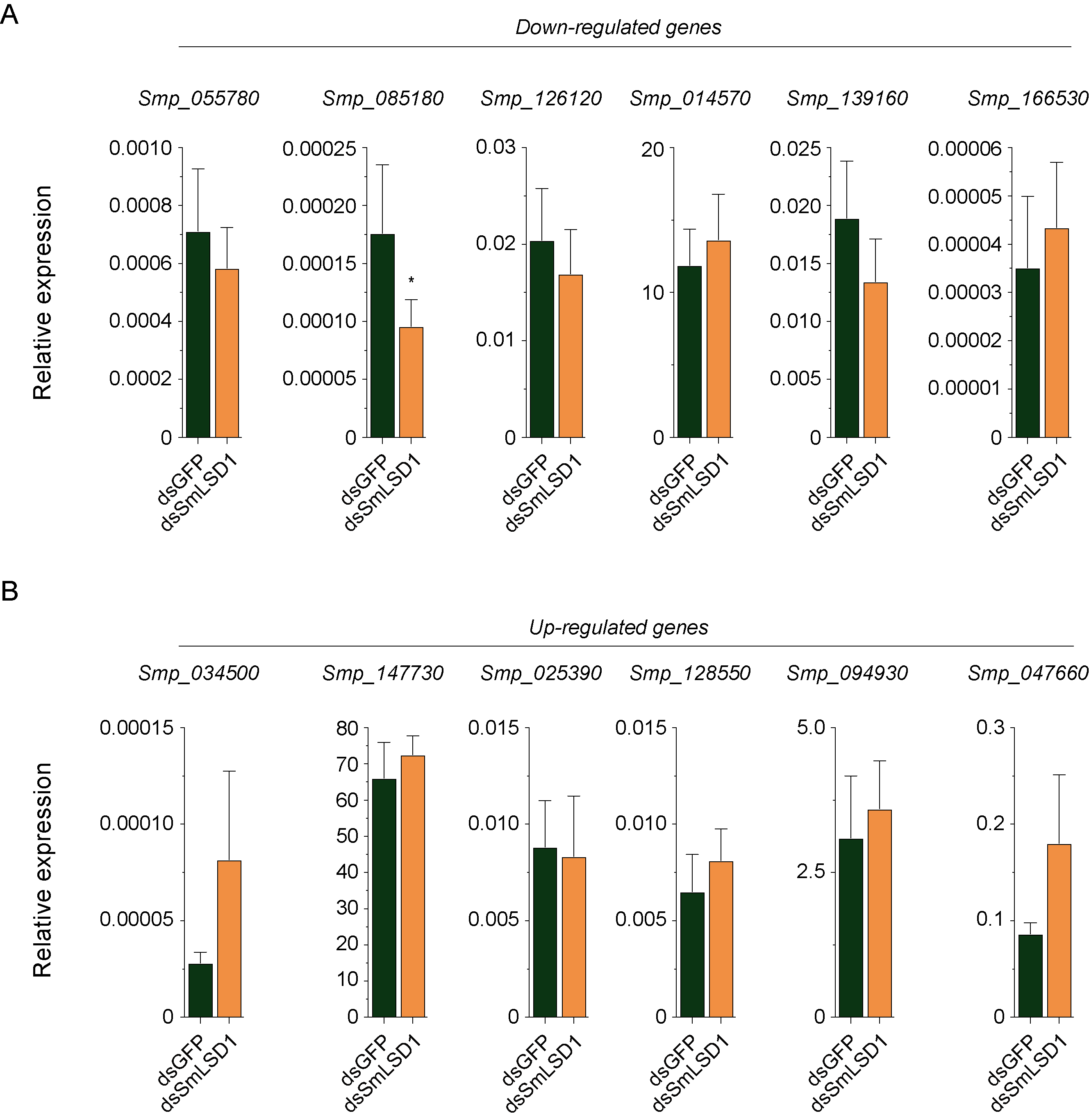

Supplement: S8 Fig — Panels A and B show the expressions of selected genes that were measured by qRT-PCR. The cDNA samples were from adult S. mansoni incubated for seven days with double-stranded RNA interference for the SmLSD1 gene or the negative control GFP gene. The methodology of the qRT-PCR and the twelve selected genes (Smp) were the same as described in S7 Fig. Statistical significance was evaluated with the Student’s t-test and significant changes are marked by asterisk with *p<0.05. (TIF) [file pntd.0008332.s008.tif]
