# Supplementary material for: Pharmacological inhibition of lysine-specific demethylase 1 (LSD1) induces global transcriptional deregulation and ultrastructural alterations that impair viability in Schistosoma mansoni
Source: PLoS Negl Trop Dis. 2020 Jul 1;14(7):e0008332. doi: 10.1371/journal.pntd.0008332 (PMC7329083; doi:10.1371/journal.pntd.0008332)
Supplement: S9 Table — (DOCX) [file pntd.0008332.s017.docx]

**Table S9**

| **Female downregulated genes** | | | | |
| --- | --- | --- | --- | --- |
| **GeneID** | **Log2FoldChange** | | | **product_description** |
|  | **Cuffdif** | **b-Sleuth** | **EdgeR** |  |
| Smp_170630 | -3,44 | -2,63 | -3,44 | Periostin 2C putative |
| Smp_085180 | -3,28 | -2,85 | -3,37 | cathepsin B (C01 family)* |
| Smp_155570 | -2,75 | -2,46 | -2,98 | endoglycoceramidase |
| Smp_055780 | -2,84 | -2,61 | -2,96 | smdr2# |
| Smp_174700 | -2,83 | -2,62 | -2,84 | transcription factor HNF 4 |
| Smp_156960 | -2,63 | -2,31 | -2,77 | nardilysin (M16 family) |
| Smp_042720 | -2,77 | -2,02 | -2,67 | beta 13 n galactosyltransferase |
| Smp_187140 | -2,58 | -2,31 | -2,65 | cathepsin L proteinase* |
| Smp_000190 | -2,56 | -2,05 | -2,64 | short chain dehydrogenase:reductase family 16C |
| Smp_147070 | -2,50 | -1,95 | -2,61 | sodium coupled neutral amino acid |
| Smp_169260 | -2,47 | -2,11 | -2,59 | zinc finger protein 362 |
| Smp_166540 | -2,42 | -2,16 | -2,56 | serine:threonine protein kinase Nek11 |
| Smp_166530 | -2,42 | -2,16 | -2,56 | phospholipase A* |
| Smp_085010 | -2,44 | -2,17 | -2,55 | cathepsin B peptidase (C01 family)* |
| Smp_075800 | -2,31 | -2,17 | -2,53 | hemoglobinase (C13 family) |
| Smp_126120 | -2,53 | -2,04 | -2,52 | LAMA protein 2 |
| Smp_036010 | -2,33 | -2,00 | -2,49 | magnesium transporter nipa2 |
| Smp_139160 | -2,53 | -2,27 | -2,48 | SmCL2 peptidase (C01 family) |
| Smp_141030 | -2,25 | -2,28 | -2,46 | epidermal growth factor receptor pathway |
| Smp_016490 | -2,42 | -2,09 | -2,42 | saposin B domain containing protein* |
| **Female upregulated genes** | | | | |
| **GeneID** | **(Log2FoldChange)** | | | **product_description** |
|  | **Cuffdif** | **b-Sleuth** | **EdgeR** |  |
| Smp_025390 | 5,76 | 3,84 | 5,77 | calcium dependent protein kinase |
| Smp_128550 | 5,13 | 3,51 | 4,92 | src type protein tyrosine kinase |
| Smp_094930 | 4,76 | 3,02 | 4,66 | early growth response protein 1 |
| Smp_134870 | 4,01 | 2,58 | 3,89 | putative early growth response protein 2 |
| Smp_124060 | 3,15 | 1,97 | 3,02 | venom allergen-like (VAL) 13 protein* |
| Smp_174810 | 3,20 | 1,93 | 2,99 | Extracellular superoxide dismutase (Cu Zn) |
| Smp_159810 | 4,15 | 2,03 | 2,91 | MEG-2 (ESP15) family* |
| Smp_172460 | 2,57 | 1,36 | 2,51 | Krueppel factor 10 like |
| Smp_049300 | 2,44 | 1,73 | 2,50 | major egg antigen 2C putative |
| Smp_186020 | 2,98 | 1,62 | 2,37 | major egg antigen |
| Smp_008660 | 2,31 | 1,48 | 2,31 | gelsolin |
| Smp_135980 | 2,25 | 1,39 | 2,16 | Calcium binding protein |
| Smp_172960 | 2,13 | 1,38 | 2,14 | serine type protease inhibitor |
| Smp_147730 | 2,13 | 1,38 | 2,14 | single kunitz protease inhibitor |
| Smp_034500 | 2,25 | 1,30 | 2,11 | Dual specificity protein phosphatase 10 |
| Smp_053560 | 2,07 | 1,29 | 2,02 | MAP kinase activated protein kinase 2 |
| Smp_123190 | 1,63 | 1,22 | 1,99 | ADP ribosylation factor protein 2 binding |
| Smp_136660 | 2,21 | 1,16 | 1,95 | pro neuregulin 2 |
| Smp_136650 | 2,21 | 1,16 | 1,95 | pro neuregulin 2 membrane bound |
| Smp_028210 | 2,02 | 1,26 | 1,89 | calcyphosin protein like |
