# Supplementary material for: Pharmacological inhibition of lysine-specific demethylase 1 (LSD1) induces global transcriptional deregulation and ultrastructural alterations that impair viability in Schistosoma mansoni
Source: PLoS Negl Trop Dis. 2020 Jul 1;14(7):e0008332. doi: 10.1371/journal.pntd.0008332 (PMC7329083; doi:10.1371/journal.pntd.0008332)
Supplement: S10 Table — (DOCX) [file pntd.0008332.s018.docx]

**Table S10**

| **Male downregulated genes** | | | | |
| --- | --- | --- | --- | --- |
| **GeneID** | **(Log2FoldChange)** | | | **product_description** |
|  | **Cuffdif** | **b-Sleuth** | **EdgeR** |  |
| Smp_180350 | -4,88 | -3,53 | -4,97 | opsin receptor |
| Smp_017620 | -4,90 | -3,39 | -4,83 | membrane primary amine oxidase |
| Smp_017610 | -4,90 | -3,39 | -4,83 | amiloride sensitive amine oxidase |
| Smp_000755 | -4,66 | -3,33 | -4,75 | family M13 non peptidase ue (M13 family) |
| Smp_135230 | -4,62 | -3,30 | -4,62 | Tyrosine DeCarboxylase family member (tdc 1) |
| Smp_160360 | -4,31 | -3,02 | -4,22 | sodium:chloride dependent neurotransmitter |
| Smp_136730 | -4,35 | -3,15 | -4,14 | cathepsin d (lysosomal aspartyl protease)* |
| Smp_149930 | -4,71 | -3,04 | -4,08 | sodium:potassium:calcium exchanger 6 |
| Smp_008610 | -4,01 | -2,91 | -4,06 | deoxyribonuclease ii |
| Smp_104890 | -3,91 | -2,79 | -4,03 | Cys loop ligand gated ion channel subunit |
| Smp_128860 | -4,03 | -2,79 | -3,99 | lysyl oxidase 2 |
| Smp_169570 | -3,95 | -2,76 | -3,94 | glycerol 3 phosphate dehydrogenase |
| Smp_170630 | -3,80 | -2,66 | -3,80 | Periostin 2C putative |
| Smp_165340 | -4,26 | -2,68 | -3,78 | alpha tocopherol transfer protein |
| Smp_143300 | -3,79 | -2,68 | -3,76 | fibrillin 1 |
| Smp_195090 | -3,71 | -2,61 | -3,75 | tegument-allergen-like protein |
| Smp_193350 | -3,69 | -2,55 | -3,63 | cadherin EGF LAG seven pass G type receptor |
| Smp_085180 | -3,77 | -2,68 | -3,59 | cathepsin B (C01 family)* |
| Smp_123780 | -3,62 | -2,58 | -3,56 | glypican 5 |
| Smp_172590 | -3,40 | -2,57 | -3,48 | family S10 unassigned peptidase (S10 family) |
| **Male upregulated genes** | | | | |
| **GeneID** | **(Log2FoldChange)** | | | **product_description** |
|  | **Cuffdif** | **b-Sleuth** | **EdgeR** |  |
| Smp_133770 | 5,71 | 3,91 | 5,68 | lengsin |
| Smp_025390 | 4,64 | 3,20 | 4,61 | calcium dependent protein kinase |
| Smp_047680 | 4,21 | 3,09 | 4,41 | ferritin 2C heavy polypeptide 1 |
| Smp_159810 | 4,65 | 2,64 | 3,86 | MEG-2 (ESP15) family* |
| Smp_128550 | 3,17 | 2,28 | 3,37 | src type protein tyrosine kinase |
| Smp_134870 | 3,21 | 2,21 | 3,14 | early growth response protein |
| Smp_172960 | 3,37 | 2,05 | 3,02 | serine type protease inhibitor |
| Smp_147730 | 3,37 | 2,05 | 3,02 | single kunitz protease inhibitor |
| Smp_172460 | 2,98 | 2,05 | 3,01 | Krueppel factor 10 like |
| Smp_048050 | 3,30 | 2,09 | 2,96 | Major egg antigen (p40) |
| Smp_051400 | 2,83 | 1,97 | 2,87 | dynein light chain |
| Smp_210990 | 2,70 | 1,62 | 2,84 | serine:threonine protein phosphatase PP1 beta |
| Smp_049230 | 3,06 | 2,01 | 2,84 | Major egg antigen (p40) |
| Smp_047650 | 2,82 | 1,95 | 2,83 | ferritin 2C heavy polypeptide 1 |
| Smp_049300 | 2,38 | 1,94 | 2,73 | major egg antigen 2C putative |
| Smp_094930 | 2,73 | 1,87 | 2,68 | early growth response protein 1 |
| Smp_129510 | 2,72 | 1,81 | 2,65 | metallophosphoesterase domain containing protein |
| Smp_130370 | 2,51 | 1,27 | 2,47 | elongation of very long chain fatty acids* |
| Smp_077880 | 2,45 | 1,73 | 2,45 | annexin |
| Smp_186020 | 2,65 | 1,56 | 2,43 | major egg antigen |
