# Supplementary material for: Pharmacological inhibition of lysine-specific demethylase 1 (LSD1) induces global transcriptional deregulation and ultrastructural alterations that impair viability in Schistosoma mansoni
Source: PLoS Negl Trop Dis. 2020 Jul 1;14(7):e0008332. doi: 10.1371/journal.pntd.0008332 (PMC7329083; doi:10.1371/journal.pntd.0008332)
Supplement: S11 Table — (DOCX) [file pntd.0008332.s019.docx]

**Table S11**

| **schistosomula downregulated genes** | | | | |
| --- | --- | --- | --- | --- |
| **GeneID** | **(Log2FoldChange)** | | | **product_description** |
|  | **Cuffdif** | **b-Sleuth** | **EdgeR** |  |
| Smp_169190 | -3,46 | -2,21 | -3,40 | tegument-allergen-like protein |
| Smp_187140 | -2,25 | -1,63 | -2,33 | cathepsin L proteinase* |
| Smp_162770 | -2,18 | -1,58 | -2,30 | lysosome associated membrane glycoprotein |
| Smp_140610 | -1,97 | -1,58 | -2,29 | iron:zinc purple acid phosphatase protein |
| Smp_085010 | -2,15 | -1,56 | -2,22 | cathepsin B peptidase (C01 family)* |
| Smp_105420 | -2,14 | -1,57 | -2,17 | Saposin%2CIPR008139 Saposin |
| Smp_028870 | -2,05 | -1,62 | -2,17 | Zinc finger%2C C2H2 type domain containing protein |
| Smp_067060 | -1,96 | -1,48 | -2,16 | cathepsin B peptidase (C01 family)* |
| Smp_166540 | -2,03 | -1,52 | -2,13 | serine:threonine protein kinase |
| Smp_105450 | -2,20 | -1,51 | -2,12 | saposin containing protein* |
| Smp_020080 | -1,91 | -1,34 | -2,00 | GTP binding protein (I) alpha subunit alpha |
| Smp_032980 | -1,94 | -1,34 | -1,95 | calmodulin protein |
| Smp_139240 | -1,87 | -1,32 | -1,81 | cathepsin S (C01 family) |
| Smp_138270 | -1,85 | -1,20 | -1,79 | diaminopimelate epimerase DafE |
| Smp_142490 | -1,64 | -1,22 | -1,77 | Transmembrane protein 45B |
| Smp_142970 | -1,77 | -1,24 | -1,76 | palmitoyl protein thioesterase 1 |
| Smp_163630 | -1,44 | -1,12 | -1,69 | developmentally regulated antigen 10 |
| Smp_152940 | -1,61 | -0,97 | -1,60 | otopetrin |
| Smp_126120 | -1,65 | -1,07 | -1,59 | LAMA protein 2 |
| Smp_212450 | -1,30 | -1,20 | -1,51 | placental protein 11 |
| **schistosomula upregulated genes** | | | | |
| **GeneID** | **(Log2FoldChange)** | | | **product_description** |
|  | **Cuffdif** | **b-Sleuth** | **EdgeR** |  |
| Smp_199840 | 0,73 | 0,48 | 0,89 | nucleolar protein c7b |
| Smp_040990 | 0,98 | 0,57 | 0,81 | Ribonuclease H2 subunit C |
| Smp_149060 | 1,00 | 0,55 | 0,80 | U3 small nucleolar RNA associated protein 22 |
| Smp_190720 | 0,78 | 0,53 | 0,66 | aspartyl tRNA synthetase |
| Smp_140560 | 0,76 | 0,45 | 0,66 | TLC domain containing protein 2 |
| Smp_092390 | 0,62 | 0,44 | 0,61 | N acetylglucosamine kinase |
| Smp_013360 | 0,57 | 0,42 | 0,57 | u1 small nuclear ribonucleoprotein 70 kDa |
| Smp_179010 | 0,64 | 0,40 | 0,56 | exosome complex component RRP42 |
| Smp_124940 | 0,70 | 0,40 | 0,56 | adrenodoxin protein 2C mitochondrial like |
| Smp_208070 | 0,58 | 0,41 | 0,54 | CDC16 cell division cycle 16 |
| Smp_002820 | 0,64 | 0,37 | 0,52 | zinc finger CCCH domain containing protein 4 |
| Smp_170030 | 0,49 | 0,35 | 0,49 | sh3 domain binding glutamic acid rich |
| Smp_022730 | 0,53 | 0,35 | 0,49 | H:ACA ribonucleoprotein complex subunit |
| Smp_042430 | 0,48 | 0,35 | 0,48 | dna replication complex gins protein sld5 |
| Smp_042420 | 0,48 | 0,35 | 0,48 | histone H4 transcription factor |
| Smp_153710 | 0,83 | 0,34 | 0,47 | mutS protein 5 |
| Smp_179160 | 0,53 | 0,41 | 0,47 | nuclear DNA binding protein |
| Smp_071640 | 0,50 | 0,33 | 0,46 | arginine:serine rich splicing factor |
| Smp_126690 | 0,55 | 0,33 | 0,45 | COP9 signalosome complex subunit 6 |
| Smp_058780 | 0,53 | 0,29 | 0,43 | leukocyte receptor cluster |
