# Supplementary material for: Pharmacological inhibition of lysine-specific demethylase 1 (LSD1) induces global transcriptional deregulation and ultrastructural alterations that impair viability in Schistosoma mansoni
Source: PLoS Negl Trop Dis. 2020 Jul 1;14(7):e0008332. doi: 10.1371/journal.pntd.0008332 (PMC7329083; doi:10.1371/journal.pntd.0008332)
Supplement: S12 Table — (DOCX) [file pntd.0008332.s020.docx]

**Table S12**

| **GeneID** | **Product description/ application** | **Foward Primer (5'-3')** | **Reverse Primer (5'-3')** |
| --- | --- | --- | --- |
| XM_018797592.1 | SmLSD1_1 | GTCGTCCCGTAACTCCAGTG | AACAGGCAAGGTTTCGGACA |
|  | SmLSD1_2 | TGTCACACGATGGAGAACTG | GAAGTGTAGATTTGTCGATTGTGAA |
|  | dsRNAi-T7/  SmLSD1_1 | GGGTAATACGACTCACTATAGGCCATCTCATACGTCGGTCCA | GGGTAATACGACTCACTATAGGCTTTCAGCAGGCGTCAGAGTA |
|  | dsRNAi-T7/  SmLSD1_2 | GGGTAATACGACTCACTATAGGGACTCGTATGTTGCTGTCGGAG | GGGTAATACGACTCACTATAGGCGGCTTCACGTAGACCACTT |
|  | qRT-PCR/SmLSD1 | CCACTTCAAACTGCCCTGTC | TCATCTTGATCCCAATGACGT |
| pEGFP-N3 | GFP | AGCAGAGCTGGTTTAGTGAACC | TTATGATCTAGAGTCGCGGCCG |
|  | dsRNAi-T7/  SmLSD1_1 | GGGTAATACGACTCACTATAGGGGGATCCATCGCCACCATGGT | GGGTAATACGACTCACTATAGGGTTACTTGTACAGCTCGTCCATGCCG |
| Smp_090920 | qRT-PCR | CACCAGCTCATCATAAATAATCCA | TAGCATCCTGAAAGCCACGA |
| Smp_062630 | qRT-PCR | GGAATGATGTGGCCGATAGT | CGCAGAGATTGGCTAAATTG |
| Smp_103140 | qRT-PCR/ SmTubulin | GGATTTGACGGAATTCCAAA | AACGCTTAACTGCTCGTGGT |
| Smp_085180 | cathepsin B (C01 family) | TATGAGTTCCCGCCGTGTAC | TGCTATGACCGGACCATTCA |
| Smp_055780 | smdr2 | TGCCTATGGTGATAATAGTCGGA | TGTGAACCATCTTGACCAGCT |
| Smp_187140 | cathepsin L proteinase | AGCTGTAACTGAATCTCAATATGCT | TGGATTGAACAACCACATCTGA |
| Smp_126120 | LAMA protein 2 | ACTGGCAATTCAAGGTTACTGG | TTGTTACTTGTGTACGGAGGTT |
| Smp_014570 | Saposin1 | TGCCGCAGATGAATCACAAT | GTGCATAACATGTTTGATCTGCT |
| Smp_139160 | SmCL2 peptidase (C01 family) | TCGAAAGGTGTTGTGAAAGTGA | ACCAACAGCTAATACACCATGA |
| Smp_166530 | phospholipase A | TCCAGCGTTCTATGCCATCT | CCAATCCATCAACAAGAGAGTGA |
| Smp_025390 | putative calcium dependent protein kinase | TCAGGAGCTACAGAGAACGT | ACTCCACGGTTTATAGCTTGT |
| Smp_128550 | src type protein tyrosine kinase | TTGCTTGGTGATACAGACGAG | GAACCAGTATCCCGAATGACC |
| Smp_094930 | early growth response protein 1 | GCAGCTCCATCTCTTTCTACTG | TCCCCACTTTGTCTAGTTGAAC |
| Smp_147730 | single kunitz protease inhibitor | CAAAGAAGAATGCGAGCGGA | ACGCTGAGTGGTTTCTGAGT |
| Smp_034500 | Dual specificity protein phosphatase 10 | TCTCCCTGCAAACGATTCAC | TGCCCCATAAATGCGAAATTC |
| Smp_047660 | ferritin%2C heavy polypeptide 1 | TGGTGGTCGTGTTCAGTACA | CGGTTAGTGCTGGATCATTGT |
