# Supplementary material for: Pharmacological inhibition of lysine-specific demethylase 1 (LSD1) induces global transcriptional deregulation and ultrastructural alterations that impair viability in Schistosoma mansoni
Source: PLoS Negl Trop Dis. 2020 Jul 1;14(7):e0008332. doi: 10.1371/journal.pntd.0008332 (PMC7329083; doi:10.1371/journal.pntd.0008332)
Supplement: Attachment — Submitted filename: RebuttalLetter_Carneiro.doc [file pntd.0008332.s025.doc]

|  | <http://images.google.com.br/imgres?imgurl=http://www.olharvirtual.ufrj.br/2006/imagens/logos/minerva.jpg&imgrefurl=http://www.olharvirtual.ufrj.br/2006/index.php%3Fid_edicao%3D188%26codigo%3D12&usg=__m69fzRgO3tEvzcckR2YYtwggGU8=&h=234&w=200&sz=13&hl=pt-BR&start=5&um=1&tbnid=uF0lsWljDC75oM:&tbnh=109&tbnw=93&prev=/images%3Fq%3Dminerva%2Bufrj%26hl%3Dpt-BR%26rlz%3D1R2GGIT_pt-BR%26sa%3DN%26um%3D1> | *Marcelo R. Fantappié, Ph.D.*  *Associate Professor*  *Institute of Medical Biochemistry*  *Federal University of Rio de Janeiro*  *Prédio do CCS, Bloco Dss - 016*  *Cid. Universitária – Ilha do Fundão*  *Rio de Janeiro - RJ - 21.941-590 - Brasil*  *Tel: +55 21 3938-6608*  *e-mail: fantappie@bioqmed.ufrj.br* |
| --- | --- | --- |
| **__________________________________________________________________________________________________________** | | |

**PLOS Neglected Tropical Diseases**

**Wei Hu – Guest Editor**

**Timothy Geary – Deputy Editor**

Rio de Janeiro, March 03, 2020

**RESPONSE LETTER**

Dear Dr. Hu and Dr. Geary,

Please find our revised manuscript entitled, Pharmacological inhibition of lysine-specific demethylase 1 (LSD1) induces global transcriptional deregulation and ultrastructural alterations that impair the viability of *Schistosoma mansoni* (PNTD-D-19-01913), by Carneiro *et al.*, which we would like to be reconsidered for publication in PNTD. Please find our point-by-point responses to the reviewers’ comments.

We would like to take this opportunity to thank the reviewers for their detailed review of our manuscript. Their input was constructive for improving our paper. We hope you will find our revised and improved work suitable for publication in PNTD.

Sincerely,

Marcelo Fantappié

**Point-by-point responses to the reviewers’ questions (in bold):**

Reviewer #1:

The figures especially 4A,B; 6; 7G,H; 8 all suffer from a lack of clarity of the labeling;

**Authors:**

**The labels in Figure 4 A and B were improved. In the revised manuscript, Panel B indicates the use of DAPI and TUNEL labeling. The legend was modified to improve clarity (page 40, lines 1050-1056). We believe Fig 6 is sufficiently clear. In all panels, the structures have been extensively labeled, and arrows, asterisks, and letters indicators are now described in the legend. The labels in Fig 7 G and H were improved, with the abbreviations of the structures better positioned. In Fig 8 A, each color of the Venn diagram represents one sample and is now properly described in the figure legend (page 41-42, lines 1091-1104). In Fig 8 B, the heat map panel was prepared based on information commonly shown in figures depicting RNA-seq analysis, with the scores for each sample presented on the right side of the map.**

Figure 1, the different areas except the white are defined;

**Authors:**

**The white areas in Fig 1 are described in the legend in the revised manuscript (page 38, lines 1011-1019).**

All the figures need to be checked.

**Authors:**

**All figures were carefully rechecked.**

Line 606 tube not tuibe:

**Authors:**

**We apologize; this error was corrected (now, line 651).**

why docking instead of crystal structure?

**Authors:**

**This is a pertinent point raised by the reviewer. In collaboration with the group of Christophe Romier at IGBMC in France, we extensively tried to obtain enough intact recombinant SmLSD1 for protein crystallization and X-ray analysis. Unfortunately, the recombinant SmLSD1 was found to be highly prone to degradation/proteolysis, despite all our efforts to change expression systems and protocols. This condition forced us to discontinue our pursuit of SmLSD1 crystallization. Nevertheless, the in silico modeling approach that we used is highly reliable.**

Reviewer #2:

Do all the compounds evaluated inhibit human (or ideally schistosome) LSD1 activity equally in vitro?

**Authors:**

**Supplementary Fig 3 A and B show the screening research from the 12 synthetic LSD1 inhibitors tested for parasite viability, and the findings revealed different toxicity levels for the schistosomula and adult worms. We did not evaluate the action of all 12 inhibitors on the SmLSD1 enzymatic activity by Western blotting. We chose the most potent inhibitor, MC3935, for this specific approach.**

Can the authors relate the IC50 for enzyme inhibition to activity against worms?

**Authors:**

**We have provided the IC_50_ for enzyme inhibition against schistosomula (Supplementary Fig 1 C) and described the related methods (page 13, lines 289-296) in the revised manuscript.**

i.e., do the most potent LSD1 inhibitors have the most potent effects on worms and vice versa?

**Authors:**

**Yes, the most potent inhibitor, MC3935, showed the most potent effects on both schistosomula and adult worms, as revealed by the results from the viability assays, optical and electron microscopy and Western blot analyses. This effect was the reason we chose MC3935 for further characterization.**

The authors report that drug treatment causes specific transcriptional changes that they attribute to changes in histone methylation. The authors also report the RNAi of LSD1 causes worms to get sick, similar to drug treatment, again attributed to less LSD1 activity. Can the authors show that RNAi of LSD1 causes similar changes in mRNA expression as drug treatment? This could provide some evidence that the effects of drugs are via LSD1 inhibition. RNAseq would be best, but qPCR could also address this point.

**Authors:**

**This is a pertinent point raised by the reviewer. We agree that RNA-seq would have been the better approach. However, performing the necessary experiment would have added another layer of complexity, time, and cost that, at this stage of the process, was deemed to be unjustifiable, especially considering the amount of data already provided in the current work. Nevertheless, following the reviewer’s suggestion, we performed qRT-PCR for RNAi validation. We also validated the results of the RNA-seq experiment that we had completed, as well as the cDNA samples from worms subjected to the same treatment as was used for RNA-seq. Although we validated nearly 100% of the genes selected (six genes upregulated and six genes downregulated) from our RNA-seq or cDNA samples (now Supplementary Fig 6 A and B), we failed to validate the differential expression of these genes in our RNAi samples, except for one gene (now Supplementary Fig 8). However, most of these genes showed a clear tendency for down or upregulation (now Supplementary Fig 8). Below, we describe a plausible explanation for the lack of correlation between the RNA-seq and RNAi data (described in the Results section, page 25-26, lines 601-617):**

*“The twelve differentially expressed genes validated by qRT-PCR from the RNA-seq analysis (Supplementary Fig S6 A and B) were tested in the samples obtained from the RNAi experiment, and we could confirm only one down-regulated gene (Smp_156960) (Supplementary Fig S8). Although curious, this was not a surprising result, if one considers that there are some caveats involved when dealing with gene silencing by RNA interference, such as partial reduction of the target mRNA levels, and partial reduction in the level of the respective protein due to its possibly low turnover rate. In the case of SmLSD1, we achieved 70 % of mRNA silencing, which still leaves 30 % of the message to be translated and possibly leaving a considerable amount of LSD1 enzymatic activity for transcriptional regulation. This phenomenon can be easily verified when comparing the levels of mono and dimethylation of histone H3 lysine 4 in the adult worm samples from MC3935 and dsRNA targeting LSD1. We found 4.16-fold increase in H3K4me1 signal in the MC3935-treated worms compared with only a 2.06-fold increase in the dsRNA treated worms. The same was observed in the H3K4me2 signal, where MC3935-treated adult worms presented a 2.76-fold increase in the histone mark whilst dsRNA treated adult worms presented only a 1.84-fold increase in this signal.”*

**Given the results of the RNA-seq analysis of MC3935 effect showing that treatment with the drug promotes a broad change in the transcriptional program of the parasite, which affected a number of different metabolic pathways, it is possible that, when selecting the 12 genes (in a somewhat arbitrary manner) for qPCR validation, we may not have included the full set of genes most relevant for the phenotypic changes observed by RNAi of SmLSD1. Nevertheless, SmLSD1 silencing was able to affect the physiology of the parasite in a similar manner when compared to the MC3935 treatment (Supplementary Fig 7E – G). This suggests that a minimal interference in the control of the parasite’s transcriptional program is already detrimental to the worm.**

**Because we could not validate the differential gene expression in our RNAi experiment, we changed the final paragraph of our Discussion (page 31, lines 724-730) as follows:**

***“****The present study further suggests SmLSD1 is a strong candidate as a drug target. More generally, the epigenetic regulation of chromatin affects many fundamental biological pathways. Therefore, the realization that the deregulation of chromatin is harmful to S. mansoni should reinforce significant efforts to develop selective epigenetic drugs against schistosomiasis. Although the results obtained do not allow us to pinpoint a specific mechanism to explain the toxicity of the inhibitor, and we cannot rule out some off-target effects.****”***

Reviewer #3:

The authors concluded that SmLSD1 is a promising drug target for the treatment of schistosomiasis. However, in the context, these effects of LSD1 inhibitor on S. mansoni has been verified only in vitro, but lack of the validation in vivo. In animals, due to complex environmental factors, such as drug absorption, etc., does it still have obvious killing effect on S. mansoni? In addition, does the drug damage animals? What’s the side effect of LSD1 inhibitor on animals? I think the manuscript should be included this part. If the LSD1 inhibitor causes severe damage to the host, this conclusion needs to be adjusted here.

**Authors:**

**This is an important issue raised by the reviewer, and we agree that the final goal of research that targets a drug candidate is validation in animals. However, we would like to emphasize that the purpose of the paper was to validate SmLSD1 as a potential target and show that promising results can be obtained with a small molecule inhibitor. We believe that we accomplished the very first step towards the final goal. The next step would be for chemists to further optimize the properties of the lead. It’s only after we design a second-generation of improvements that in vivo effects can be ascertained.**

**Nevertheless, an initial attempt towards this aim was initiated through collaboration with Kancera, a Sweden pharmaceutical company (www.kancera.com). Kancera performed in vitro chemical evaluations (of purity, stability, solubility, and the formulation) and stage 1 pharmacokinetics (PK) studies of MC3035. The results, although still preliminary, showed that 50 mg/kg MC3935 administered p.o. appeared to be well tolerated by mice. However, the plasma concentration of MC3935 was quite low. Since we have shown that MC3935 is very potent against *S. mansoni* *in vitro*, the low plasma levels may provide enough exposure to elicit an effect. On the other hand, the free fraction level of the compound in the plasma is not yet known.**

**Similarly, the question about human versus worm selectivity in terms of inhibition is also to be addressed in the optimization phase. In addition, there is no correct answer: an equal inhibition of the schistosome and a human enzyme may be irrelevant for the use of an inhibitor as a medicine. There are many antimicrobial drugs that are not particularly selective. Therefore, the studies on the action of MC3935 in animals are still very preliminary. Nevertheless, we are committed to follow up with in vivo research and thus have consistently applied for support for such a study and expect that our proposal will eventually secure the necessary funding.**

Line 285-286: This part about adult worm motility and egg laying assay should use the paired adult worms, but not mixing with paired or unpaired.

**Authors:**

**We thank the reviewer for making this observation, which indicated that our description led to misinterpretation. The sentence describing the related methods was altered accordingly. Briefly, every experiment described in this article was based exclusively on paired adult worms. For the viability assay, based on ATP dosage, we prepared a total cell lysate of every treated adult worm, independent of their paired state. The very first alteration observed after LSD1 inhibitor treatment was the unpairing of coupled adult worms. Thus, the viability of the collectively cultured parasites was used for ATP quantification.**

What is the statistical method used in the article? Please add in the part of method.

**Authors:**

**The significance of all pertinent assays was determined using Student’s t-tests, with * p<0.05, ** p<0.01, *** p<0.001 and **** p<0.0001. This information was added to all the figure legends.**

How many parasites have been observed in each group in figure 5, 6 and 7? Are all the same in each group?

**Authors:**

**In our scanning, transmission, and confocal microscopy experiments, we analyzed fifteen parasites. It was essential to highlight that damage was identified (under the microscopies) in 100% of the samples treated with MC3935. All the samples treated with DMSO presented with healthy structures.**
