# Supplementary material for: Pharmacological inhibition of lysine-specific demethylase 1 (LSD1) induces global transcriptional deregulation and ultrastructural alterations that impair viability in Schistosoma mansoni
Source: PLoS Negl Trop Dis. 2020 Jul 1;14(7):e0008332. doi: 10.1371/journal.pntd.0008332 (PMC7329083; doi:10.1371/journal.pntd.0008332)
Supplement: Attachment — Submitted filename: reviewers comments-0405.docx [file pntd.0008332.s026.docx]

Schistosomiasis mansoni is a chronic and debilitating tropical disease caused by the helminth Schistosoma mansoni. The control and treatment of the disease rely almost exclusively on praziquantel (PZQ). Thus, there is an urgent need to search for promising protein targets to develop new drugs. This study is meaningful and interesting, but only i*n vitro* test has been performed. Since there are still many uncertainties, the significance of LSD1 inhibitor should not be exaggerated, and the conclusion should be made carefully.

The font format in Line 601 and below is definitely not the same as the above.

Which statistical software used in the article? Student t test was used when comparing, but the author did not mention which was the control? For example, in Fig 2, Untreated or DMSO? And the statistical analysis method should be included in the method section. And the t value and p value should be given in the text.

In scanning, transmission, and confocal microscopy experiments, the number of parasites analyzed should be included in the figure legend and method section.
